# Hyaluronidases as Targets for the Treatment of Neurological Diseases

**DOI:** 10.1002/pgr2.70036

**Published:** 2025-09-23

**Authors:** Larry S. Sherman, Barbara A. Sorg, Steven Matsumoto, Weiping Su, Fatima Banine, Stephen A. Back

**Affiliations:** 1 Division of Neuroscience, Oregon National Primate Research Center, Oregon Health & Science University, Beaverton, Oregon, USA; 2 Department of Cell, Developmental & Cancer Biology, Oregon Health & Science University, Portland, Oregon, USA; 3 Program in Neuroscience, Washington State University, Vancouver, Washington, USA; 4 R.S. Dow Neurobiology, Legacy Research Institute, Portland, Oregon, USA; 5 Department of Biomaterial and Biomedical Science, School of Dentistry, Oregon Health & Science University, Portland, Oregon, USA; 6 Department of Neurology, Oregon Health & Science University, Portland, Oregon, USA; 7 Department of Pediatrics, Oregon Health & Science University, Portland, Oregon, USA

**Keywords:** alcohol use disorder, brain injury, CEMIP, hyaluronidase, multiple sclerosis, stroke

## Abstract

Hyaluronan (HA) is a glycosaminoglycan synthesized at the cell membrane that can exist in numerous states in the extracellular matrix, including in ternary complexes with proteoglycans such as aggrecan and neurocan. HA synthesis is elevated following a wide variety of insults to the central nervous system (CNS) including neuroinflammatory disease, ischemia, and various forms of dementia. Recent studies have demonstrated that, in conjunction with increased HA synthesis, the expression and activities of hyaluronidases that digest HA are also elevated in the injured CNS. While high molecular weight forms of HA have their own functions that can be disrupted by hyaluronidases, digestion products of HA generated by these hyaluronidases have their own, distinct biological activities that can impact recovery from CNS damage. Here, we review some of the conditions and diseases in which hyaluronidase activity can play a role in preventing CNS repair and discuss the potential ways that hyaluronidase inhibitors could be used as therapeutic agents.

## 1 | Introduction

A major component of the central nervous system (CNS) extracellular matrix (ECM) is hyaluronan (HA), a glycosaminoglycan composed of unbranched, non-sulfated repeating units of glucuronic acid and *N*-acetyl-glucosamine. In the CNS, HA is found in diffuse ECM, which is soluble in saline and detergents, as well as in condensed ECM, which forms aggregates, including proteoglycan-rich perineuronal nets (PNNs) [1, 2].

HA is synthesized by transmembrane HA synthases (called HAS1, HAS2, and HAS3) that are widely expressed in varying combinations and levels by mammalian cells. These glycosyltransferases synthesize the (→4)-β-d-GlcUA-(1 → 3)-β-d-GlcNAc (1→) disaccharide repeats of HA by repetitive addition of the monosaccharide units from uridine diphosphate-activated (UDP) donors (reviewed in [3]). HAS enzymes utilize UDP-*N*-acetylglucosamine (GlcNAc) and UDP-glucuronic acid (GlcA) precursors in the cytoplasm, whose levels may, in part, determine the amount and size of the final HA molecule that is formed. Indeed, different HAS enzymes can generate HA molecules ranging in size from ≤ 2.5 × 10^5^ Da to high molecular weight (HMW) sizes ≥ 4 × 10^6^ Da. These different HMW forms of HA have numerous biological activities that include regulating cell migration, proliferation, and differentiation [2, 3].

HA catabolism is highly regulated with approximately a third (5 g) of the body’s total HA content turning over each day [4]. In mammals, HA is catabolized by a variety of hyaluronidases that digest HA at the β1,4 linkage. There are six hyaluronidase-like genes (*HYALs*; Table 1), found on human chromosomes 3 and 7. These genes include *HYAL1, HYAL2*, and *HYAL3* found at 3p21.3 and *HYAL4, SPAM1* (*PH*-*20*), and *HYALP1* at *7q31*.3. These hyaluronidases share approximately 40% nucleotide sequence homology [4]. *HYALP1* is an expressed pseudogene in humans, while the function of HYAL3 as an independent hyaluronidase is unclear. Interestingly, mice lacking Hyal3 do not demonstrate HA accumulation in their tissues and overexpression of HYAL3 in cells can induce increased HYAL1 activity, suggested that HYAL3 functions by facilitating HYAL1 [5, 6]. *HYAL4* is expressed at high levels in the placenta and skeletal muscle in humans, but only at low levels in other tissues [7], and appears to digest chondroitin and not HA [8]. *SPAM1* (*PH*-*20*) is found in the testis under normal physiological conditions, although it has also been reported to be detectable in fetal and placental cDNA libraries and in other cells under pathological conditions [7]. Another hyaluronidase gene, *Hyal5*, has also been identified but is only found in rodents [4]. Hyal1, Hyal2, Hyal4, Hyal5, and PH20 act as endo-β-N-acetyl-hexosaminidases that cleave the β1–4 glycosidic linkages in HA through a catalytic domain that is highly conserved between the different enzymes.

HYAL1 and HYAL2 have hyaluronidase activity in somatic tissues. HYAL1 is associated with lysosomes and has a pH optimum of 3.7. It can hydrolyze endocytosed HA molecules with a wide range of sizes [9]. Like HYAL1, HYAL2 has been reported to have lysosomal localization but is also present within lipid rafts of the cell membrane, where it can associate with the CD44 transmembrane HA receptor [10]. HYAL2 cleaves large HA into much smaller (around 20 kDa) fragments and has a pH optimum of 6.0–7.0 [11]. A model for how HYAL2 functions suggests that it forms an extracellular acidic pocket via interaction of CD44 with the Na+/H+ exchanger at the cell membrane [10]. HA catabolism may therefore involve the initial extracellular digestion of HA by HYAL2, PH20, or other extracellular HA catabolizing proteins at extracellular pockets, followed by further lysosomal digestion of HYAL1 within cells.

In addition to the HYALs and PH20, other proteins with HA catabolizing activity have been identified including the cell migration-inducing and HA-binding protein (CEMIP) [12] and transmembrane protein 2 (TMEM2; also called CEMIP2) [13], although how these proteins catabolize HA is unclear. While the structures of these proteins are distinct from the HYALs, CEMIP, and TMEM2 share 48% amino acid identity with one another [4]. Given the fact that these proteins can both facilitate the digestion of HA, either directly or indirectly, we will consider their roles in HA digestion along with the HYALS and PH20.

**FIGURE 1 |:**
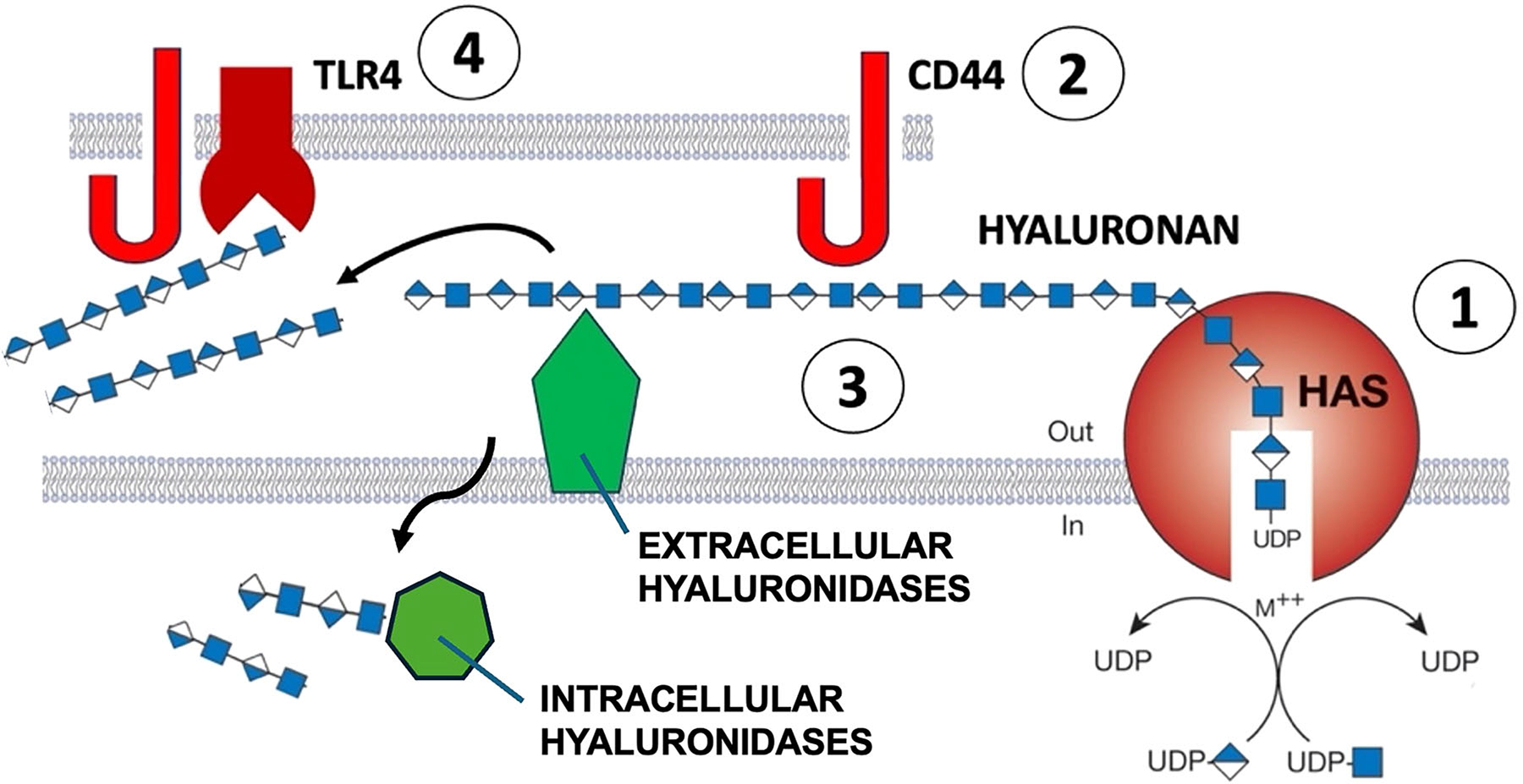
(1) HA is synthesized at the cell membrane by transmembrane HA synthases (HAS1, HAS2, and HAS3) that link uridine diphosphate (UDP) to alternating molecules of N-acetylglucosamine and glucuronic acid. Growing HA chains are extruded into the extracellular matrix where they form secondary and tertiary structures on their own or associate with proteoglycans. HMW HA is recognized by CD44 (2) or other HA receptors, either on the same cell or adjacent cells, and is catabolized by extracellular hyaluronidases or HA-catabolizing proteins, such as GPIanchored HYAL2, CEMIP, or TMEM2 (3), as well as intracellular hyaluronidase activity (e.g., HYAL1) that is typically in lysosomes. These intracellular hyaluronidases digest HA fragments that are internalized by receptor-mediated endocytosis, resulting in smaller HA fragments that become substrates for exoglycosidases. Extracellular HA fragments generated by hyaluronidases have their own biological activities that can be mediated by CD44 in conjunction with other receptors, such as toll-like receptor-4 (TLR4) (4).

Although the mechanisms by which CEMIP and TMEM2 cause HA digestion are unclear, several findings give clues about how they function. Some studies have supported a mechanism of HA depolymerization by CEMIP that involves localization of CEMIP in clathrin-coated vesicles followed by internalization into early endosomes and excretion of degraded HA molecules into the extracellular space [12]. CEMIP-mediated HA depolymerization may also require interactions with Annexin-1 at the cell membrane [14]. Other studies have suggested that the activity of CEMIP may involve its inclusion in exosomes [15]. While CD44 may help localize HA at the cell membrane, it does not appear that CD44 is required for CEMIP-mediated HA depolymerization. On the other hand, digestion products of HA, including fragment sizes that are generated by CEMIP, can influence cellular signaling by interacting with CD44 and other transmembrane receptors, including Toll-like receptors 2 and 4 [16, 17] (Figure 1).

TMEM2 is expressed as a transmembrane protein and shares significant homology with CEMIP. Although there have been several studies that suggest TMEM2 may not be directly involved in HA catabolism (e.g., [18, 19]), *tmem2*-mutant zebrafish demonstrate HA accumulation [20]. Furthermore, inducible *Tmem2* knock-out mice demonstrate elevated levels of HA in the serum, liver, lung, kidney, skin, and bone marrow, supporting a role for Tmem2 in systemic HA turnover [21]. One recent study demonstrated that the ectodomain of TMEM2 from humans and mice degrades both fluorescein-labeled and native HA into 5−10 kDa fragments [22].

Numerous studies have suggested that HA catabolism is increased following a variety of insults to the CNS [3]. These studies suggest that either the disruption of HMW HA or the generation of specific sizes of HA digestion products can impact CNS injury and recovery. In this review, we discuss several examples of how HA catabolism may impact CNS diseases and injuries. We further suggest ways that regulating HA catabolism could provide unique therapeutic interventions for a variety of conditions where HA catabolism prevents CNS repair.

## 2 | Hyaluronidases in Demyelinating Diseases

Numerous studies have implicated changes in HA synthesis and catabolism in the pathogenesis of demyelinating lesions. Myelin insulates the axons of neurons and dramatically increases their conduction velocities. Conditions where myelin fails to form (e.g., leukodystrophies or perinatal brain injury) or conditions that cause demyelination (e.g., multiple sclerosis) lead to conduction block or conduction delay, causing motor, sensory, and cognitive dysfunction. In the CNS, myelin is generated by oligodendrocytes (OLs), which arise throughout life from oligodendrocyte progenitor cells (OPCs). One way that remyelination can fail following demyelination is through the inhibition of OPC maturation into myelinating OLs [23].

HA accumulates in both experimental and clinical demyelinating lesions, including lesions from patients with multiple sclerosis [24–29]. Several studies reported that CD44 is expressed by OPCs and that the addition of HMW HA to cultures of OPCs blocks OPC maturation [25, 30–33], while injecting HMW HA into demyelinating lesions delays remyelination [25, 30, 32–34]. It was therefore suggested that the HA that accumulates in demyelinating lesions could itself block OPC maturation and remyelination [25]. Subsequent studies, however, found that there is hyaluronidase activity within demyelinating lesions [17, 30, 34]. At least some of this activity appears to come from OPCs themselves that generate specific sizes of HA fragments that block OPC maturation and remyelination. Furthermore, blocking hyaluronidase activity in OPC cultures promotes OPC differentiation into OLs, and remyelination and increased axon conduction velocities in experimentally induced demyelinating lesions [31–34].

Consistent with these findings, HA digestion products generated by PH20 were found to block OPC maturation and remyelination, and some reports suggested that *PH20* is expressed, albeit at barely detectable levels, in demyelinating lesions and by OPCs in vitro [28, 34]. However, subsequent reports failed to detect PH20 in either MS or mouse lesions [33, 35]. Another report suggested that CEMIP is expressed by astrocytes in MS demyelinating lesions [36]. However, a more recent study failed to find CEMIP in astrocytes but did demonstrate that OPCs, but not mature OLs, express CEMIP and that CEMIP-digested HA can block OPC maturation and remyelination [33]. Furthermore, inhibitors that block CEMIP activity promote OPC maturation, remyelination, and increased conduction velocities in demyelinated lesions [31–33].

Neuronal activity is also impacted in demyelinating diseases as a result of both the loss of myelin and neuroinflammatory insults that impact neurons. Interestingly, *Cemip* knock-out mice demonstrate spatial memory impairments that are similar to those observed in *Cd44* knock-out mice [37, 38]. These changes in memory function occur coincident with HMW HA accumulation in the hippocampus. The mechanism of these deficits could be related to at least transient alterations in myelination during development but myelination delay has not been examined in either Cd44- or Cemip-null animals. Alterations in spatial memory function in these mice could also be related to decreased numbers of doublecortin-positive immature neurons in the dentate gyrus of the hippocampus, where *Cemip* mRNA is highly expressed in wild-type mice [37, 39]. In addition, dendritic spine density is significantly decreased in the dentate gyrus granule cells in *Cemip* knock-out mice [39]. These findings are also consistent with a recent study reporting that hyaluronidase causes simultaneous membrane depolarization and calcium influx into neurons [40]. CEMIP-mediated HA digestion may therefore be important for synapse formation. Given that HA digestion in the dentate gyrus by another hyaluronidase can induce increased neural stem cell proliferation and the accumulation of doublecortin-positive immature neurons [41], CEMIP may also be a critical regulator of adult hippocampal neurogenesis.

Although the role of TMEM2 in demyelination is unclear, one study suggested that reducing TMEM2 expression in OPCs could ameliorate the effects of HA on OPC maturation [42]. It is unclear, however, if these effects were due to hyaluronidase activity. The study also did not explore whether blocking TMEM2 could promote remyelination.

Collectively, these studies support the hypothesis that demyelinating insults lead to a combination of increased HA synthesis along with increased expression of one or more hyaluronidases by OPCs and possibly other cells, including neurons and neural stem cells, in the microenvironments of demyelinating lesions. The resulting elevated hyaluronidase activity generates HA fragments that block OPC maturation into OLs, thus preventing remyelination, while also disrupting neurogenesis and neuron activity. Hyaluronidase inhibitors could, therefore, contribute to therapies that promote remyelination.

## 3 | Hyaluronidases in Stroke

Ischemic stroke is the leading cause of neurological disability in adults and a leading cause of death [43]. Ischemic lesions progress over time and, like demyelinating lesions, include substantial neuroinflammation, neuronal and glial cell death, and changes in the ECM that contribute to secondary damage and poor recovery. HA synthesis and catabolism have been implicated in stroke outcomes for many years. For example, in a 2013 study using a mouse model of ischemic brain injury, Has2 and Hyal2 protein expression were elevated in peri-infarct tissue in the subventricular zone and rostral migratory stream [44]. Similarly, *Hyal1*, *Hyal2*, and *Has2* mRNAs become upregulated in peri-infarct tissues in a rat model of stroke induced by occlusion of the middle cerebral artery [45, 46]. Elevated HAS1, HAS2, HYAL1, and HYAL2 were also found in post-mortem brain tissue of stroke patients [47], and elevated HYAL1 was observed in serum from stroke patients [47].

One study tested the hypothesis that blocking hyaluronidase activity following strokes could lead to improved clinical outcomes. These authors examined the effects of oral administration of the broad-spectrum hyaluronidase inhibitor, l-ascorbic acid 6-hexadecanoate (VCPAL), in mice following photothrombotic stroke in somatosensory cortex [48]. As in previous studies, they observed upregulation of mRNAs of genes encoding HA synthesizing and degrading enzymes in the perilesional area at early times after stroke, and immunostaining showing increased Hyal1 in astrocytes and Has2 in astrocytes and neurons. Animals treated with VCPAL at these early times demonstrated improved performance on a skilled reaching test [48]. These results are consistent with the hypothesis that after stroke, hyaluronidase exacerbates the progression of peri-infarct damage and that blocking this activity can aid in stroke recovery, although other mechanisms cannot be ruled out. It will be interesting to explore whether CEMIP is also elevated following stroke, and whether CEMIP or HYAL1 influences HA catabolism at different times following stroke. Additional studies will be required to confirm that the effects of VCPAL on stroke recovery are entirely due to blocking hyaluronidase activity, and to determine the specific hyaluronidases involved in promoting secondary CNS damage.

## 4 | Hyaluronidases in Perinatal Brain Injury

Preterm birth is associated with a high risk of brain injury. Brain damage in preterm infants can result from hypoxiai-schemia (HI), infections, chronic hypoxia, and treatments often used following premature births such as mechanical ventilation and corticosteroids. Many of these insults can cause significant inflammation that further contributes to infant morbidity and mortality.

Substantial evidence supports the hypothesis that HA synthesis and catabolism may influence the outcomes of perinatal brain injuries. A study of archival tissues from preterm human infants with white matter injury reported diffuse astrogliosis in lesions surrounded by elevated HA and high expression of CD44 [49]. These areas were accompanied by increased numbers of lateappearing pre-OLs, consistent with the findings in adult forms of demyelinating diseases where an expanded number of pre-OLs populate lesions but fail to differentiate [50]. A preterm sheep model of HI also resulted in diffuse white matter injury with an expanded population of pre-OLs that failed to differentiate into myelinating OLs [51]. In preterm fetal sheep that sustained recurrent HI (rHI), white matter injury was accompanied by an expanded population of preOLs concomitant with elevated levels of HA and increased expression and transcription of PH20 within tissues, although this expression was not subsequently observed in postnatal white matter, and other hyaluronidases were not examined [28].

Seizures are common in preterm newborns and can worsen any initial brain injury linked to prematurity. Infants who develop seizures typically have poor neurodevelopmental outcomes. Interestingly, apigenin, a natural flavonoid that blocks hyaluronidase activity [52], was reported to have antiseizure and neuroprotective effects in the kainic acid-induced seizure model in mice and rats [53]. Apigenin also suppressed spontaneous seizure spikes 1 month after kainic acid-induced status epilepticus [54]. A study in a sheep model of preterm brain injury explored the possibility that blocking hyaluronidase activity could affect the development or severity of seizures [55]. This study treated animals with apigenin or S3, a novel hyaluronidase inhibitor previously shown to promote remyelination as described above [32]. Infusion of apigenin or S3 both reduced the number of animals with seizures, total seizure time, and mean seizure burden [55]. S3 treatment was also associated with a reduction in the total number of seizures, while apigenin was associated with earlier cessation of seizures. Neither the earlier rodent studies using kainic acid nor the study of perinatal brain injury-related seizures in sheep examined how apigenin or S3 influenced HA levels in the brains of treated animals. Nonetheless, while it is possible that both S3 and apigenin share activities other than blocking hyaluronidase activity, these findings collectively suggest that hyaluronidase activity is a target to reduce perinatal seizures following ischemia.

## 5 | Hyaluronidases in Traumatic Brain Injury (TBI)

TBI continues to be a leading cause of mortality in children and young adults. Like ischemic injuries, TBI is characterized by initial tissue damage followed by subacute progressive damage that develops in response to the initial injury. One study examined the expression of HA synthases, hyaluronidases, and HA receptors in rats subjected to controlled-cortical impact-induced TBI (CCI) [56]. Compared to naïve controls, *Has1* and *Has2* mRNA, but not *Has3* mRNA, increased significantly following craniotomy alone and following CCI. *Hyal1* mRNA expression also increased significantly in the craniotomy group and in the contralateral CCI group, while *Cd44* mRNA expression increased significantly in the ipsilateral CCI group. It was unclear from this study if increased HA synthesis or *Hyal1* expression was linked to the progression of secondary damage.

One of the sequelae of TBI is cerebral edema that can cause significant secondary damage. A study investigated the possibility that alterations in HA synthesis and catabolism could influence cerebral edema in mice following TBI through the so-called Gibbs-Donnan effect, where a porous, negatively charged matrix can attract positive ions and water [57]. The study found that intracerebroventricular injection of bovine testicular hyaluronidase, whose activity is largely PH20, reduced edema in the ipsilateral hippocampus following CCI. Hyaluronidase did not adversely affect blood-brain-barrier-integrity as measured by dynamic contrast-enhanced magnetic resonance imaging, or functional recovery after CCI [57]. A subsequent study found that Tmem2 expression increased in a rat TBI model, and that reducing Tmem2 expression worsened injury outcomes [58]. It is unclear if the hyaluronidase activity of Tmem2 could be attributed to these effects. Nonetheless, these data support the hypothesis that increased hyaluronidase activity in different models of TBI can have protective effects, especially with regard to the onset of edema at early times after injury. What remains unclear is the degree to which prolonged hyaluronidase activity could impact CNS repair at later times following TBI once that edema has resolved.

## 6 | Potential Roles for Hyaluronidases in Alzheimer’s Disease (AD) and Vascular Dementia

AD, vascular dementia, and related neurodegenerative diseases affect millions of people worldwide. Although the roles of hyaluronidases in the progression of these diseases are unclear, there is increasing evidence that dysregulated HA synthesis and catabolism may impact different aspects of AD and other dementias. Multiple studies have shown that HA accumulates in the brain with normative aging, largely through the actions of increased HA synthase activity [59, 60]. In human AD cases and in mouse models of AD, this HA accumulation is increased in multiple brain regions, especially in areas where amyloid plaques and tau tangles are present due, at least in part, to elevated HAS1 or HAS2 expression [61, 62]. In the white matter of AD cases with vascular brain injury, HA accumulates in white matter coincident with increased numbers of OPCs, suggesting that HA may impact OPC maturation in ways that are similar to those observed in MS [63]. It is unclear, however, if hyaluronidase activity is also elevated in these areas where HA synthesis is increased.

Among the risk factors for late-onset AD is disruption of the endolysosomal pathway that leads to impaired clearance of proteins, including cytotoxic variants of amyloid beta [64]. Interestingly, one study found that HYAL1 reversed endolysosomal dysfunction following exposure to cytotoxic amyloid-beta in a neuronal cell culture model through a CD44-dependent mechanism [65]. Digested HA also attenuated neurotoxicity in treated neurons, suggesting that the effects of HYAL1 involved the generation of HA fragments that impacted cell survival [65]. These authors then tested if elevated HYAL1 could influence disease progression by injecting a lentivirus carrying the *HYAL1* gene into the dentate gyrus of 6- to 7-month-old 3xTg-AD mice that bear three human genes associated with familial AD: APP^KM670/671ML^ (Swedish), MAPT^P301L^, and PSEN1^M146V^. After 5 weeks, mice expressing elevated HYAL1 demonstrated improved cognitive function [65]. It is possible, therefore, that HA fragments generated by hyaluronidase activity within neurodegenerative lesions could have neuroprotective activity. Future studies will be required to assess the possibility of using hyaluronidases for this purpose and to determine the mechanisms by which they influence AD and other dementias, but also if hyaluronidase activity could at the same time have a negative impact on OPC maturation in the white matter of individuals with dementias.

## 7 | HA Catabolism Following Heavy Drinking

Alcohol use disorder (AUD) is a chronic relapsing disorder associated with excessive drinking. Data from humans and from a nonhuman primate model of AUD suggest that heavy drinking leads to changes in the composition of the ECM in multiple brain areas linked to mediating excessive alcohol drinking and impaired cognitive flexibility [66]. This finding is consistent with the hypothesis that the ECM could influence the activity of cells in the CNS that impact drinking behaviors or responses to drinking.

One way that alcohol-related changes in ECM can impact AUD is through the tetrapartite synapse which is composed of neuronal pre- and post-synaptic membranes, astroglial processes, and ECM. HA is a major component of this ECM, and alterations in tetrapartite synapse activity have been implicated in drinking behaviors [67]. In addition to the interstitial ECM of the tetrapartite synapse, HA is also a key component of PNNs, both in its soluble form and in forms associated with proteoglycans. PNNs form during development and are specialized net-like structures that surround mostly parvalbumin+ (PV), fast spiking GABAergic neurons and proximal dendrites, with openings for synaptic inputs [68]. PNNs are dynamic in adulthood and are widely implicated in regulating learning and memory and changes in neuron function following CNS injury [69–72]. PV neurons profoundly inhibit the network of surrounding neurons via elaborate contacts with pyramidal neurons [73] and strongly contribute to the excitatory:inhibitory (E:I) balance essential for normal functioning [74]. Not only do PV neurons powerfully control the output of pyramidal PV neurons, in the medial prefrontal cortex (PFC), they are critical for cognitive flexibility tasks that rely on new strategies during ruleshifting [75–77].

PNNs have been implicated in both responses to drinking and in drinking behaviors. In general, ethanol exposure increases PNNs and PNN components in cortical regions [78], although PNNs can also decrease in number depending on the drinking paradigm and the brain regions examined (e.g., [78–80]). One study [81] used an adolescent binge model of ethanol exposure via intermittent intragastric administration for 6 days and reported an increase in the staining density of PNNs as well as their components, the HA-associated proteoglycans brevican and neurocan, in the orbitofrontal cortex. More recent adolescent binge ethanol exposure studies in rats demonstrated increases in the number of PNNs around PV neurons in cortical regions, including the orbitofrontal and medial PFC [82] and anterior insular cortex in rats with concurrent impairment in behavioral flexibility on an attentional set-shifting task [83]. Using a different model of binge drinking (drinking in the dark; DID), Chen et al. [84] found similar increases in PNN staining as well as in PNN components, including aggrecan, brevican, and phosphacan in the insular cortex 1 day following 6 weeks of DID. This effect was not observed 1 week later, supporting the notion that bingelike drinking causes dynamic changes in the ECM comprising PNNs. Such dynamic changes are likely due to alterations in the synthesis or catabolism of components of the ECM, including HA.

**FIGURE 2 |:**
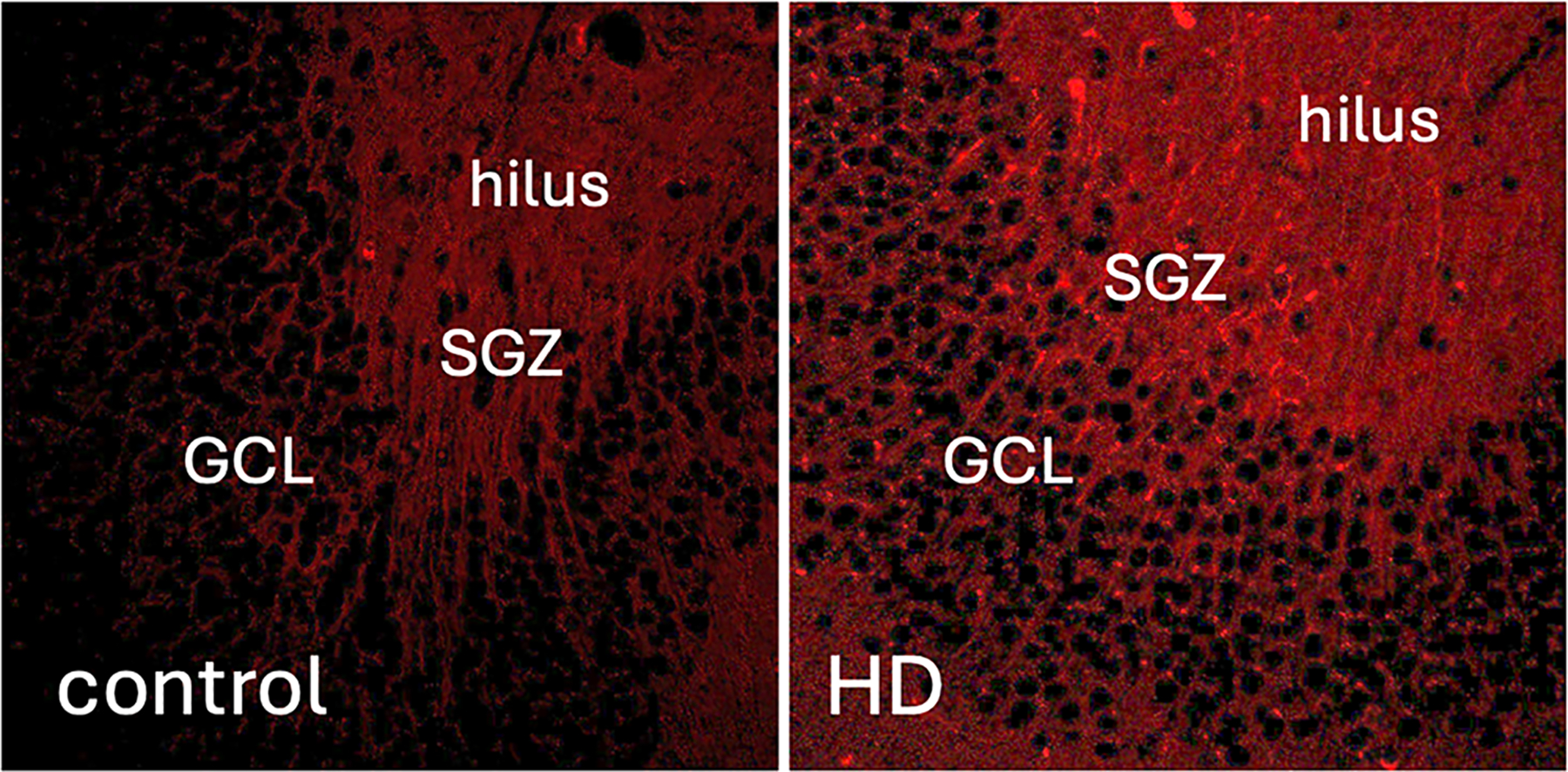
Changes in HA levels as measured by staining with a biotinylated HA-binding protein (red) in the DG of (left panel) control (non-heavy drinkers) versus (right panel) heavy drinking (HD) rhesus macaques. In this drinking protocol, animals (4−6 years old) go through an induction phase of 4 months of schedule-induced polydipsia with 16 h of drinking sessions followed by an “open access” phase of 6 months with 22 h/day of voluntary drinking sessions (4% ethanol w/v solution or water choice). HD animals consumed > 4.0 g/kg/day of ethanol. Note the increased HABP labeling in the HD tissue section. GCL = granule cell layer, SGZ = subgranular zone.

Patients with advanced AUD demonstrate elevated levels of HA in their blood and livers compared to patients without AUD [85, 86]. Consistent with this finding, ethanol induced *Has2* transcription and increased hepatic HA in mice [87]. Using a well-established model of drinking in rhesus macaques [88], we found that HA staining in the hippocampal dentate gyrus is increased following long-term drinking with periods of abstinence (Figure 2). It is possible that HA catabolism is initially elevated in the brain during early periods of drinking but that HA synthesis increases or hyaluronidase activity decreases either with extended drinking or during periods of abstinence. Such changes in HA synthesis and catabolism could account for alterations in PNNs and other areas of CNS ECM during heavy drinking and could impact the activity of neurons that regulate drinking behaviors.

## 8 | Clinical Implications

The studies reviewed here suggest that hyaluronidases are elevated in lesions associated with a wide variety of CNS disorders. In some cases, blocking hyaluronidase activity can have beneficial effects that include promoting remyelination, improving outcomes following strokes, and blocking seizures following perinatal brain injuries. These effects may involve a combination of blocking the degradation of HMW HA and the generation of fragments of HA that have activities that are distinct from HMW HA. On the other hand, elevated hyaluronidase expression and activity may have beneficial effects, including regulating edema following TBI [57] and neuroprotective effects in AD and other dementias [65]. Thus, any therapeutic strategy that utilizes hyaluronidase inhibitors will require a full understanding of both the positive and negative impacts of blocking hyaluronidase activity, and consideration for the type of injury where blocking hyaluronidase activity is most appropriate.

Given that elevated hyaluronidase activity can play multiple negative roles in CNS injury and disease, it will be important to develop potent and specific hyaluronidase inhibitors that can cross the blood-brain-barrier and which have limited side effects for both preclinical studies and for clinical applications. Such agents could lead to novel therapeutic strategies for a wide range of conditions and diseases. Some of the best characterized hyaluronidase inhibitors include polyphenols, especially flavonoids, such as apigenin [89]. A recently described flavonoid inhibitor of CEMIP, sulfuretin, has potential utility in both promoting neuroprotection and remyelination [31]. In addition, phenolic acids, tannins, and other chemicals have also shown varying degrees of hyaluronidase inhibitory activities [89]. Interestingly, heparin and dextran sulfate, a synthetic sulfated polysaccharide, can potently inhibit CEMIP [90]. However, most of these agents have limited specificity for one hyaluronidase over others or have additional activities unrelated to hyaluronidases.

In addition to the roles played by hyaluronidases in the CNS, increased hyaluronidase expression and activity have also been implicated in numerous other diseases including brain tumors and other cancers [91]. Given their roles as tissue spreading factors, hyaluronidases have also been explored as targets to treat exposures to snake and other venoms by limiting their dispersion through tissues [92]. Future studies will be needed to identify inhibitors that are highly specific and have limited side effects to use as therapeutic agents for CNS and other diseases in which hyaluronidase activity promotes disease progression.

A common finding in CNS injuries and diseases is that HA synthases and different hyaluronidases simultaneously demonstrate elevated expression in the same tissues (e.g., [44–47]). This observation suggests that there is an interplay between HA synthesis and catabolism that reflects the need to tightly regulate the accumulation of HMW HA. A result of HA synthases and hyaluronidases being co-expressed is that lower MW forms of HA that have their own biological activities through different receptors are generated, which could have detrimental effects in the injury microenvironment when there is excess HMW HA as seen in demyelinating lesions [17]. It is also possible that under certain circumstances at different times following a CNS injury that these lower MW HA molecules have beneficial effects as observed in an experimental model of AD [65]. It will be important to explore the distinct roles of these hyaluronidasegenerated products in each of these different insults.

In addition to the putative roles for hyaluronidases in brain injuries linked to specific diseases and trauma, the finding that HA is dysregulated in the dentate gyrus following heavy drinking raises the exciting possibility that manipulating HA catabolism could be beneficial for recovery from AUD through mechanisms that involve regulating neuron activity. Future studies will determine whether the changes in HA observed in the dentate gyrus following heavy drinking are also observed in other brain regions that are affected by ethanol consumption. It will also be interesting to assess if these changes in HA are due to decreased HA catabolism or increased HA synthesis, and if alterations in hyaluronidase activity specifically impact PNNs and the activity of PV neurons.

The changes in PNNs observed in heavy drinking are not unique to individuals with AUD. Addiction to other substances, including cocaine, may also depend on altered PNN ECM throughout the brain [93]. Changes in PNNs have also been implicated in altered neuronal activity in other conditions, including multiple sclerosis [94], ischemic brain injuries [95], TBI [96], and seizures [97]. Drugs or other agents that impact hyaluronidase activity may therefore function at least in part by regulating PNNs in each of these conditions.

## 9 | Conclusions

Altogether, the findings reviewed here suggest the exciting possibility that targeted manipulation of specific hyaluronidases could have multiple benefits ranging from promoting nervous system repair to influencing neuronal activity through the regulation of PNNs. It will be important to determine at what stages following any given insult hyaluronidase activity is beneficial or detrimental, which hyaluronidases should be targeted, and how long any therapeutic approach is safe and efficacious. Nonetheless, pharmacological or other approaches that regulate hyaluronidase activity hold substantial promise for the treatment of neurological disorders.

## Figures and Tables

**TABLE 1 |:** Human HA catabolizing enzymes and proteins.

Name	HA catabolizing activity?	Expression in normal tissues	Expression in normal or injured CNS?
HYAL1	Yes	Widely expressed	Yes
HYAL2	Yes	Widely expressed	Yes
HYAL3	Unclear	Widely expressed	Yes
HYAL4	No	Placenta and skeletal muscle	No
HYAL5 (rodents only)	Yes	Sperm	No
HYALP1	No	Expressed in several tissues	No
PH20	Yes	Sperm	Unclear
CEMIP	Yes	Widely expressed	Yes
TMEM2	Yes	Widely expressed	Yes

## Data Availability

The authors have nothing to report.

## Abbreviations:

AD: Alzheimer’s disease
AUD: alcohol use disorder
CCI: controlled-cortical impact-induced TBI
CEMIP: cell migration-inducing and HA-binding protein
CNS: central nervous system
DID: drinking in the dark
ECM: extracellular matrix
E:I: excitatory: inhibitory ratio
GABA: gamma aminobutyric acid
HA: hyaluronan
HAS: hyaluronan synthase
HMW: high molecular weight
HYAL: hyaluronidase
H-I: hypoxia-ischemia
OL: oligodendrocyte
OPC: oligodendrocyte progenitor cells
PFC: prefrontal cortex
PNN: perineuronal net
PV: parvalbumin
SPAM1: sperm adhesion molecule-1
TBI: traumatic brain injury
TLR: toll-like receptor
TMEM2: transmembrane protein-22
